# Targeting Oncogenic Signaling in Mutant FLT3 Acute Myeloid Leukemia: The Path to Least Resistance

**DOI:** 10.3390/ijms19103198

**Published:** 2018-10-16

**Authors:** Dilana Staudt, Heather C. Murray, Tabitha McLachlan, Frank Alvaro, Anoop K. Enjeti, Nicole M. Verrills, Matthew D. Dun

**Affiliations:** 1 School of Biomedical Sciences and Pharmacy, Faculty of Health and Medicine, University of Newcastle, Callaghan, NSW 2308, Australia; Dilana.StaudtBarreto@uon.edu.au (D.S.); Heather.C.Murray@uon.edu.au (H.C.M.); Tabitha.McLachlan@uon.edu.au (T.M.); nikki.verrills@newcastle.edu.au (N.M.V.); 2 Priority Research Centre for Cancer Research, Innovation & Translation, Faculty of Health & Medicine, Hunter Medical Research Institute, New Lambton Heights, NSW 2305, Australia; Frank.Alvaro@hnehealth.nsw.gov.au (F.A.); Anoop.Enjeti@calvarymater.org.au (A.K.E.); 3 John Hunter Children’s Hospital, Faculty of Health and Medicine, University of Newcastle, New Lambton Heights, NSW 2305, Australia; 4 Calvary Mater Hospital, Hematology Department, Waratah, NSW 2298, Australia; 5 NSW Health Pathology North, John Hunter Hospital, New Lambton Heights, NSW 2305, Australia

**Keywords:** acute myeloid leukemia, FLT3, tyrosine kinase inhibitors, resistance

## Abstract

The identification of recurrent driver mutations in genes encoding tyrosine kinases has resulted in the development of molecularly-targeted treatment strategies designed to improve outcomes for patients diagnosed with acute myeloid leukemia (AML). The receptor tyrosine kinase FLT3 is the most commonly mutated gene in AML, with internal tandem duplications within the juxtamembrane domain (FLT3-ITD) or missense mutations in the tyrosine kinase domain (FLT3-TKD) present in 30–35% of AML patients at diagnosis. An established driver mutation and marker of poor prognosis, the FLT3 tyrosine kinase has emerged as an attractive therapeutic target, and thus, encouraged the development of FLT3 tyrosine kinase inhibitors (TKIs). However, the therapeutic benefit of FLT3 inhibition, particularly as a monotherapy, frequently results in the development of treatment resistance and disease relapse. Commonly, FLT3 inhibitor resistance occurs by the emergence of secondary lesions in the *FLT3* gene, particularly in the second tyrosine kinase domain (TKD) at residue Asp835 (D835) to form a ‘dual mutation’ (ITD-D835). Individual FLT3-ITD and FLT3-TKD mutations influence independent signaling cascades; however, little is known about which divergent signaling pathways are controlled by each of the FLT3 specific mutations, particularly in the context of patients harboring dual ITD-D835 mutations. This review provides a comprehensive analysis of the known discrete and cooperative signaling pathways deregulated by each of the FLT3 specific mutations, as well as the therapeutic approaches that hold the most promise of more durable and personalized therapeutic approaches to improve treatments of FLT3 mutant AML.

## 1. Introduction

Acute myeloid leukemia (AML) is characterized by the malignant transformation of a hematopoietic stem/progenitor cell (HSC). This occurs following the acquisition of somatic driver mutations that cooperate with accrued passenger mutations, or lesions that coincidentally occur following the acquisition of the driver mutations [1]. The malignant precursor cells accumulate in the bone marrow and blood at the expense of healthy blood cells, leading to acute symptoms including anemia, bleeding and bruising, infections, and bone pain.

AML is the most common form of acute leukemia in adults and the second most common leukemia in children [2]. It is generally considered a disease of the elderly, with a median age at diagnosis of 67 years [3]. Elderly patients who are unable to receive intensive chemotherapy are only predicted to survive for 5 to 10 months post diagnosis [4]. Although AML only accounts for 20% of children diagnosed with leukemia, it is responsible for over half of all pediatric leukemia deaths [5]. The disease can emerge as a primary (*de novo* AML), a pre-leukemia (such as myelodysplastic syndrome—MDS), or can be induced following chemotherapy, radiation therapy, immunosuppressive therapy, or a combination of these used to treat pre-existing conditions [6].

Advances in genomic sequencing techniques and technologies have identified recurrent mutations which have begun to help elucidate the complex genomic landscapes underpinning the disease, both at diagnosis and following relapse [7,8]. Importantly, these studies have begun to associate individual mutations, and combinations of mutations, with overall survival (OS) [9]. Whole genome sequencing analysis has revealed that mutations are common in signaling genes that encode for the tyrosine kinases, *FLT3*, *JAK2*, *cKIT*, for phosphatases, *PTPN11*, *PTPRT*, *PTPN14*, and for Ras GTPases, *KRAS* and *NRAS*, and represent 59% of all gene mutations [8,9]. These are often independently associated with poor outcomes [10]. The commonality of these mutations, particularly to tyrosine kinases, make them attractive molecular targets; however, as it stands, targeting individual mutations using precision therapies has failed to deliver the anticipated increased survival.

**Figure 1:**
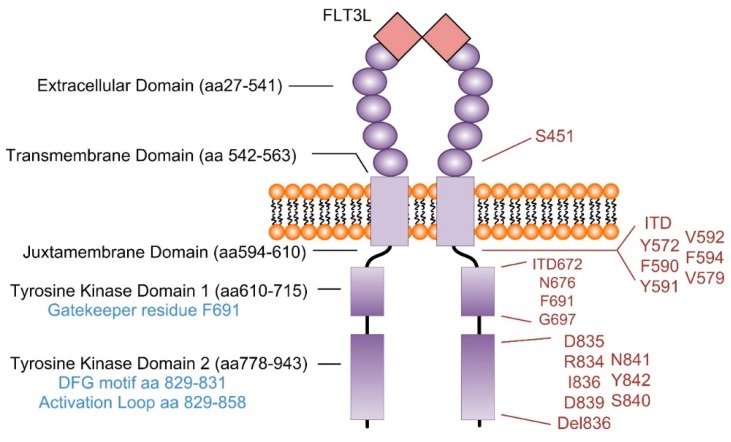
Domains and AML-associated mutations of the FLT3 receptor tyrosine kinase. The FLT3 kinase is a 993 amino acid protein comprised of extracellular, transmembrane, juxtamembrane, and split kinase domains. Mutations which have been reported in AML are notated on the right-hand side of the figure. Figure created using data from references [11,14,20,21,22,23,24,25,26,27,28,29].

Oncogenic mutations to the FMS-like tyrosine kinase 3 (FLT3) receptor occur in 30–35% of all AML cases at diagnosis [8,9]. Mutations in FLT3 can manifest as a duplication of a fragment within the juxtamembrane domain coding region (exons 14 and 15), causing an internal tandem duplication (FLT3-ITD) [11], or as missense mutations in the activation loop (exon 20) of the tyrosine kinase domain (FLT3-TKD), most commonly at aspartic acid 835 (D835; Figure 1) [12]. The presence of FLT3-ITD mutations at diagnosis is predictive of a poor prognosis, associated with increased risk of relapse and reduced overall survival [13,14]. The prognostic significance of FLT3-TKD is, however, more complex, with reports of adverse effects [13,15], no effect [16,17], and favorable prognosis [18]. This discrepancy could be attributable to differences in mutant allelic burden [19].

Despite promising initial results, FLT3 inhibitors have shown non-durable anti-leukemic activity as single-agents, with progression seen in virtually all patients [30]. In general, the durability of response tends to be only weeks to months, with long-term efficacy compromised by primary or secondary acquired resistance [31,32]. Frequently, drug resistance emerges through the development of FLT3-ITD point mutations [33] or TKD mutations [34] following treatment with tyrosine kinase inhibitors (TKIs). Recent clinical trials have demonstrated increased disease-free survival (DFS) with FLT3 inhibitors in combination with chemotherapy [35], suggesting that combined targeted strategies are required for sustained FLT3 inhibition. Indeed, in 2017, the FDA approved the FLT3 inhibitor midostaurin in combination with standard of care chemotherapy for the treatment of newly-diagnosed FLT3-mutant AML patients [36], superseding an era of very little advancement in AML therapy [37].

Despite the recurrence of FLT3-activating mutations in AML, the differences in oncogenic pathways in patients harboring either ITD or TKD mutations still require clarification. The significant advancements recently made in unbiased, quantitative phosphoproteomic profiling [38,39,40,41] have the potential to provide us with a full annotation of the signal pathways deregulated in response to individual mutations, or following acquired resistance induced by ‘dual lesions’ (e.g., *FLT3*-ITD-TKD) [42]. Herein, we review the known oncogenic signaling pathways modulated in FLT3 mutant AML, and the roles that each of the different FLT3 mutations play in the emergence of targeted-therapy resistance following the use of first and second generation TKIs. A fundamental understanding of the unique and redundant signaling pathways associated with each individual, or dual mutations, holds promise for the development of long-lasting therapeutic approaches to treat these poor prognosis AML patients.

## 2. Genomics Underpinning Transformation in AML

Myeloid malignancies are rarely heritable, usually emerging from sporadic acquired somatic mutations originating in hematopoietic progenitors [43,44]. More than 2% of healthy individuals carry several genetic alterations which are characteristic of a hematologic malignancy in the genome of their hematopoietic cells [45]. The presence of AML-associated mutations in genes such as *IDH1*, *IDH2*, *DNMT3A*, *TET2*, *SRSF2*, and *TP53* are associated with increased likelihood of developing AML later in life. *FLT3* and *NPM1* mutations are not among the baseline mutations which have been observed, and as such, are likely later events in leukemogenesis [46,47]. As in many other cancer types, leukemogenic evolution can take many years, a process known as the “pre-leukemic phase” [48,49]. Transformation to AML is characterized by a two-hit model of pathogenesis, where class I mutations confer proliferative advantages, and class II mutations impair hematopoietic differentiation and/or induce the acquisition of self-renewal properties [44,50]. This process follows for a specific evolutionary trajectory compounding several events, each of them generating a small cluster of new mutations, though only one or two are potentially pathogenic [7,9]. Early phase mutations affect genes involved in epigenetic regulation (i.e., *DNMT3A*, *TET2*, *IDH1/2*, and the cohesin complex) and prevent cell differentiation [49]. Later phase mutations are cooperating driver lesions (e.g., *FLT3*-ITD or *KRAS*) that induce full transformation into a malignant leukemic founding clone, which will give rise to subsequent subclones [7,8]. Over time, the coexistence of multiple ancestral and emerged clones creates the clonal heterogeneity responsible for disease progression [7,51].

## 3. *FLT3*-ITD Mutations Confer a Poor Prognosis in Cytogenetically Normal (CN) AML

Chromosomal alterations are one of the most common characteristics of cancer [52]; however, these events are only seen in 30–35% of AML patients, while 65–70% of patients display a normal karyotype [7,8,53]. Patients harboring ≥3 chromosome aberrations in the absence of t(8;21)(q22;q22), inv(16)(p13q22)/t(16;16)(p13;q22) and t(15;17)(q22;q21), form a separate category known as AML with a complex karyotype (CK-AML) [54]. CK-AML is a marker of prognosis, classifying patients into poor-risk groups [7,55]. However, individual gene rearrangements and point mutations are not sufficient to cause AML alone; multiple acquired mutations must occur to convert normal HSCs into leukemic stem cells (LSCs) [50,56]. This is particularly the case for cytogenetically normal (CN-AML) patients who were previously assigned an intermediate-risk prognosis in the MRC UK risk stratification [57,58].

FLT3-ITD mutations are rarely seen at diagnosis in patients harboring CK-AML [54,59]; however, they occur in 30–40% of patients with CN-AML [60,61]. Presence of a FLT3-ITD mutation is a predictor of poor prognosis in CN-AML [55]. FLT3-ITD mutations tend to co-occur with mutations in genes related to DNA methylation (*DNMT3A*, *TET2*, *IDH2*), histone methylation (*NPM1*, *MLL3*), myeloid transcription factors (*RUNX1*, *WT1*), and signaling mediators (*FLT3*-TKD) [62]. Among these, DNMT3A and NPM1 are the most common [8]. Importantly, the mutations co-associated with FLT3 have been demonstrated to hold prognostic significance. FLT3-ITD occurs in combination with NPM1 mutations in 40% of CN-AML [63], with the presence of an NPM1 mutation associated with improved prognosis compared to FLT3-ITD alone. This is recognized by the European LeukaemiaNet risk prognostic stratification of AML. Patients with high FLT3-ITD allelic ratio (>0.5) in the presence of wild-type NPM1 are classified as being of adverse risk, but are at intermediate risk if NPM1 mutated [55]. Similarly, patients with a low FLT3-ITD allelic ratio (0.05–0.5) and wild-type NPM1 are of intermediate prognostic risk, while with mutant NPM1 confers a favorable risk profile, although these patients still experience inferior survival to other favorable-risk patients [55].

Conversely, oncogenic co-operation is observed between FLT3-ITD and DNMT3A mutations (13.3% of CN-AML) to confer anunfavorable outcome [64,65]. Concurrent mutations in FLT3, NPM1, and DNMT3A are associated with heavy disease burden, and poor treatment response, event-free survival (EFS), and OS [8,9,66,67]. This is probably a bystander event, with NPM1 not adding to the prognosis in the presence of the other two driver mutations. Co-occurrence of MLL-partial tandem duplication (*MLL*-PTD, otherwise known as *KMT2A*) with FLT3-ITD also confers a poor prognosis (25% of CN-AML) [14,68]. The presence of mutations affecting genes encoding epigenetic modifiers such as DNMT3A, IDH1/2, and TET2 possibly induces the genomic instability which is responsible for the induction of FLT3-ITD mutations, converting leukemic cells into resistant clones able to undergo clonal expansion [69].

Patients with FLT3-TKD represent approximately 5–7% of all AML cases, and 11% of CN-AML [12,70]. FLT3-TKD mutations again occur most frequently with NPM1 mutations (10–15%) [63], but unlike FLT3-ITD mutations, are not consistently associated with inferior survival [14,71]. However, in concert with MLL-PTD, FLT3-TKD confers an extremely poor prognosis [9]. The emergence of additional TKD mutations in FLT3-ITD AML patients (*FLT3*-ITD-TKD) represents an important mechanism of resistance following use of TKIs [31,34,72,73,74], leading to a very poor prognosis. Following the same pattern of co-occurrence as single mutants, dual mutant FLT3 tends to co-occur with DNMT3A and NPM1, and is negatively associated with TP53 and NRAS mutations [8,9]. Functional characterization of mutant FLT3 and the modifying effect of co-operative mutations will be crucial for the identification of improved treatment strategies for each molecular subtype of AML.

## 4. Signaling Pathways Regulated in FLT3 Mutant AML

FMS-like Tyrosine Kinase 3 (FLT3; also known as CD135) is a membrane-bound receptor tyrosine kinase (RTK) which localizes to the cell surface following post-translational glycosylation [75]. FLT3 belongs to the type III RTK family, which comprises five immunoglobulin-like extracellular domains, a transmembrane domain, and intracellular juxtamembranous and split kinase domains (Figure 1). All five members of the type III RTK subclass (KIT, PGDFRα, PDGFRβ, CSFR, and FLT3) have critical roles in normal hematopoiesis regulating proliferation, differentiation, and survival [76].

**Figure 2:**
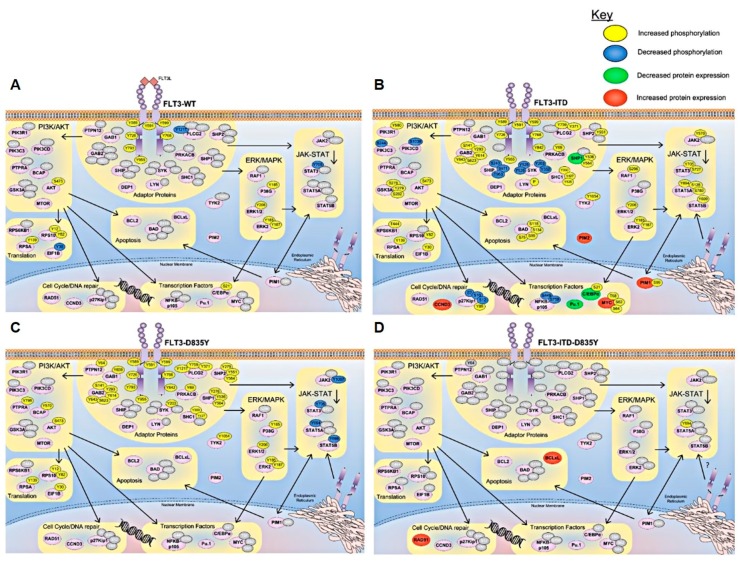
Canonical FLT3 and mutant FLT3 signaling pathways. Similar and divergent signaling pathways of wild-type and mutant FLT3 identified by functional genomic studies and large scale proteomic profiling experiments. (**A**) Upon binding of the FLT3 ligand (FLT3L), the FLT3 receptor undergoes a change in conformation and trans-autophosphorylation. (**B**) FLT3-ITD, (**C**) FLT3-TKD and (**D**) FLT3-ITD-TKD dual mutations lead to constitutive, ligand independent activation of the FLT3 receptor. Subsequent recruitment of adaptor proteins effects the activation of downstream kinase signaling pathways, such as MAPK, JAK-STAT, and PI3K. Coloring indicates activation of pathway phosphorylation or expression changes associated with the wild-type and mutant FLT3 receptor forms. Numbers indicate amino acid residues of the human protein sequence. Figure created using data from references [18,72,99,100,101,102,103,104,105,106,107,108,109,110].

FLT3 is activated by its endogenous ligand (FLT3 ligand; FLT3L) in an autocrine and paracrine manner, in synergy with interleukin-3 (IL-3), stem cell factor, or other cytokines [75] (Figure 1 and Figure 2). Binding of FLT3L leads to dimerization of the FLT3 receptor and a conformational change in the intracellular tyrosine kinase domains, exposing phosphoryl acceptor sites [77,78]. Subsequent trans-autophosphorylation of FLT3 leads to binding of adaptor proteins such as SHP2, Grb2, and Src family kinases [79,80], inducing activation of downstream kinase signaling pathways including MAPK [81], STAT [82], and PI3K [83], driving cell growth and survival.

FLT3 is predominantly expressed by hematopoietic progenitor cells [84], with expression lost or reduced during differentiation into mature lymphoid and myeloid cells [85,86,87]. The majority of AMLs express the FLT3 receptor and its ligand [88]. FLT3-ITD and FLT3-TKD mutations disrupt the auto-inhibitory resting conformational state of the receptor, inducing constitutive activation of FLT3 and ligand-independent cell growth [12,89,90]. The expression of the FLT3-ITD mutation in mouse models is sufficient for the development of fatal myeloproliferative neoplasm [91,92,93], although an extra genetic “hit” is required for the development of acute leukemia [94,95,96]. While not as well-characterized, FLT3-TKD mutations also induce myeloproliferative disease in mice, but with a less aggressive phenotype compared to FLT3-ITD carriers [97,98].

The cellular pathways differentially activated by FLT3-ITD and FLT3-TKD mutants in comparison to FLT3 wild-type have been largely characterized in mouse models and in relation to canonical FLT3 proliferation and survival signaling pathways. FLT3-ITD and FLT3-TKD lead to activation of similar pathways to the wild-type ligand-stimulated receptor, with some divergences (Figure 2). Both FLT3-ITD and FLT3-TKD mutants display increased retention in the endoplasmic reticulum, from where they display differential phosphorylation patterns and kinase activity [102,111] which may contribute to divergent signaling. FLT3-TKD is associated with increased activation of SHP1 and SHP2 phosphatases [100]; SHP1 in particular negatively regulates JAK signaling [112]. Consistently, FLT3-TKD mutants display low levels of JAK2 and STAT3 activation (Figure 2). In contrast, FLT3-ITD mutants are associated with a high level of JAK/STAT signaling (Figure 2), including phosphorylation of STAT5A; which is not induced by the wild-type receptor [104]. This suggests that increased JAK/STAT signaling is at least one mechanism by which FLT3-ITD cells may confer an oncogenic advantage over FLT3-TKD mutant activated cells; indeed, JAK inhibitors have been shown to elicit synergistic cell death in combination with FLT3 inhibition in FLT3-ITD AML cells in vitro [113]. Additionally, FLT3-ITD expression is associated with decreased protein expression of transcription factors Pu.1 and C/EBPα [104], which may contribute to impaired cell differentiation.

Currently, there is a limited number of studies characterizing signaling pathways activated downstream of FLT3-ITD-TKD dual mutations. FLT3-ITD-D835Y retains the ability to activate STAT5A [104], and is also associated with increased expression of DNA repair protein RAD51 and anti-apoptotic protein BCLxL, which may contribute to drug resistance and enhanced cell survival [72]. Further proteome-wide studies characterizing mutant FLT3 signaling in human samples are warranted.

## 5. Relapse and Resistance is Common in *FLT3*-ITD AML

The cornerstone 7 + 3 therapy for AML at diagnosis achieves remission rates of 63% [114] and is followed by additional intensive consolidation chemotherapies [115,116,117]. For intermediate and poor prognosis patients (including FLT3 mutant patients) that achieve complete remission (CR), allogeneic hematopoietic stem cell transplant (alloHSCT) leads to the highest probability of long-term survival [118,119].

Although more than half of all AML patients achieve initial CR, the majority will relapse [58,120], with expected survival for relapsed patients approximately 10% [121]. The poor outcomes following relapse are dictated mostly by the biological heterogeneity of the disease, which includes a series of patient and disease-associated factors. Refractory disease and relapse usually results from the clonal evolution of leukemic cells which gives rise to biologically distinct, and increasingly drug resistant, malignant populations [4,7]. Cytotoxic chemotherapies induce DNA damage, thus promoting the acquisition of additional mutations which may alter cell growth and drug sensitivity [7,62,122]. Furthermore, chemotherapy itself contributes in the selection of pre-existing and treatment-induced drug resistant clones that will outgrow and drive relapse [123].

Patients relapsing early after achieving CR (<1 year) most likely relapse due to a chemotherapy-resistant disease, whereas disease displaying initial responsivity to chemotherapy followed by relapse may be due to the presence of quiescent, chemotherapy-resistant leukemic stem cells (LSCs; CD34+/CD38−/CD123+ cells) [124,125]. Patients with high proportions of LSC display significantly lower relapse free survival (RFS) rates compared to those with less LSC [125,126]. Like AML blast cells, LSCs require mutations in genes encoding signaling proteins and transcription factors to promote overt AML [48,127]. Furthermore, the dormancy of LSCs confers higher chemo-resistant than proliferating AML blast cells, making LSC characterization an important research endeavor [123,128].

FLT3-ITD mutant AML patients carry CD34+/CD38−/CD123+ LSCs in the bone marrow that uniformly harbor ITD mutations. FLT3-ITD mutant LSCs are also common in patients diagnosed as FLT3 wild-type, who then relapse with FLT3-ITD mutant AML. The presence of FLT3-ITD in LSCs is associated with expression of CD123 [128,129], encoding the IL3 receptor subunit alpha (IL-3RA) which is overexpressed in 45% of AML patients. FLT3-TKD mutations, on the contrary, are not associated with high fractions of CD34+/CD38− cells [129]. LSC harboring FLT3-ITD support the notion that this mutation is a driving event in leukemogenesis, and may contribute to the high propensity of relapse in FLT3-ITD AML. The presence of LSCs with FLT3-ITD mutations is associated with poor clinical outcomes [130,131]. This justifies efforts to track LSCs, which may have clinical utility in monitoring minimal residual disease (MRD) [132].

Characterization of chemotherapeutic resistance afforded by LSCs is an important area of research, particularly in the context of MRD monitoring; however, the low frequency of LSCs makes this a challenging endeavor. Instead, characterization of LSCs using AML cell line models has gone some way to revealing the intracellular enzyme activities associated with the ‘stemness’ of LSCs. LSC cell line models harboring FLT3-ITD mutations are yet to be developed. However, the Kasumi-1 AML cell line, which possesses a mutation in another type III RTK, cKIT, (CD34+, cKIT-N822K), is considered a valuable model for the study of LSC properties. Kasumi-1 cells show upregulated *CEBPA*, *BMI-1* and *NOTCH-1* gene expression [133], which may provide protection to these primitive cells from traditional and precision therapies through altered cellular differentiation.

Ultimately, the identification of models to study LSCs harboring FLT3-ITD mutations or complex cytogenetics will offer the best hope of characterizing the oncogenic signaling that may afford LSC specific targeting in high-risk or poor prognosis AML patients. However until appropriate models can be developed, the rarity of these cells precludes unbiased proteome-wide analysis.

## 6. FLT3 Targeted Therapy

### 6.1. Tyrosine Kinase Inhibitors in Clinical Development for AML

**Figure 3:**
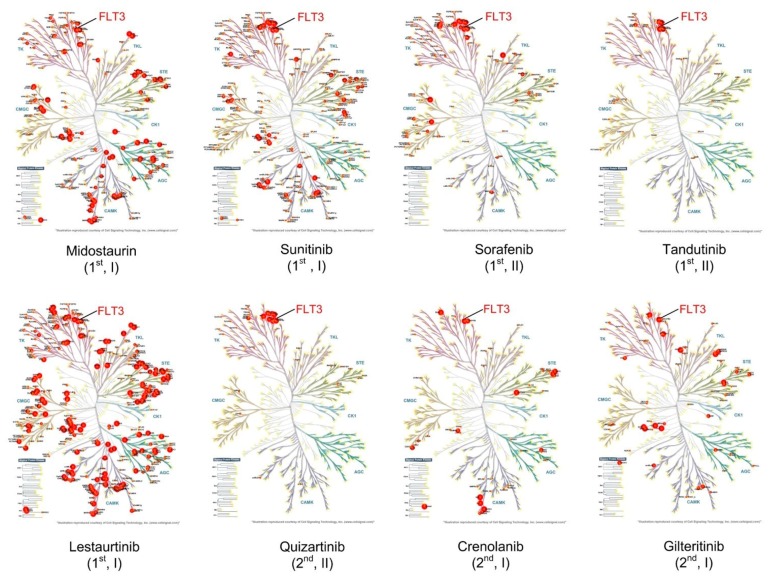
Kinase targets of first- and second-generation FLT3 inhibitors. Kinase trees depicting the dissociation constant (*Kd*, (nM)) of the target kinases of each inhibitor. Size of red bubble is proportional to the *Kd* value, with a larger bubble indicating a lower *Kd*. First generation inhibitors = 1st, second generation inhibitors = 2nd. Brackets indicate whether the inhibitor is type I or type II. Illustrations reproduced Courtesy of Cell Signaling Technology, created using KinMap [138], with data from references [139,140,141].

One of the first TKIs developed for clinical use, the BCR-ABL inhibitor imatinib, revolutionized the therapeutic landscape for chronic myeloid leukemia (CML) patients. Since the clinical introduction of TKIs for CML therapy in 2001 [134], 10-year survival rates have improved from 20% to over 80% [135,136]. Following this, there have been many attempts to develop TKIs to replicate this striking response in other malignancies driven by constitutive kinase activation, including the development of FLT3 TKIs for AML. However, despite initial favorable responses, the majority of clinical trials for FLT3 TKI monotherapy have seen the development of treatment resistance and relapse in less than 3 months of therapy. Combination therapeutic approaches are returning promising results, but the challenge remains to identify which patients will respond. Second generation FLT3 inhibitors offer highly-potent and specific FLT3 inhibition compared to first generation FLT3 inhibitors (Figure 3); however, it remains to be determined whether this translates into increased clinical benefit. Resistance to each FLT3 TKI is associated with a different profile of FLT3 mutations (Table 1). The ATP-competitive FLT3 TKIs are designated either type I or type II dependent on the mechanism of FLT3 inhibition; type I inhibitors bind the active form of the kinase that is associated with a “DFG-in” (Asp-Phe-Gly-DFG motif at the N terminus of the activation loop) conformation. Type II inhibitors bind the “DFG-out” (conformation that is only accessible when the kinase is inactive, Figure 1). As FLT3 mutations affect the conformation of the receptor, the sensitivity of FLT3 mutants towards TKI varies between the different activating mutations present [137] (Table 1 and Table 2).

####  6.1.1. First-Generation TKIs

*Midostaurin* (*PKC412*): Midostaurin is an oral, multi-targeted TKI that promotes direct and indirect inhibition of mutant FLT3 receptor phosphorylation. Midostaurin has been shown to induce cell cycle arrest and apoptosis in both FLT3-ITD and FLT3-D835Y mutant cell lines with an IC50 of less than 10 nM [148]. Midostaurin was also effective in a panel of FLT3-ITD-TKD mutant lines, although TKD1 mutations may confer some resistance [149] (Table 1 and Table 2). In a phase II study of midostaurin monotherapy, 14 out of 20 patients with relapsed/refractory AML or myelodysplastic syndrome with FLT3 activating mutations achieved approximately 50% reduction in peripheral blasts [150]. In a phase IIB study of 95 patients with relapsed/refractory AML or myelodysplastic syndrome irrespective of FLT3 status, 50 mg or 100 mg midostaurin administered twice daily showed acceptable tolerability and high rates of blast reduction, with one FLT3-ITD+ patient achieving partial remission (PR) [151]. A phase III placebo-controlled study followed, using induction and consolidation chemotherapy regimens combined with midostaurin, and followed by one year maintenance with midostaurin in 717 patients with newly diagnosed FLT3 mutated AML (ITD or TKD) [35]. Despite no significant difference in rates of CR, patients treated with midostaurin achieved significantly longer EFS and median OS. Improved OS in patients with low (0.05–0.7) and high FLT3-ITD mutant allelic burden using midostaurin suggests that the therapeutic mechanism of action may not be solely due to FLT3 kinase inhibition, but may include inhibition of multiple kinases (Figure 3). This highlights the potential benefit for use in FLT3 wild-type and FLT3-ITD patients. This study led to FDA approval of midostaurin in combination with chemotherapy in newly-diagnosed AML patients with mutated FLT3, providing the first hope for realization of a precision therapy in AML.

*Sorafenib* (*BAY 43-9006*): Sorafenib is an oral multi-targeted TKI, FDA approved for treatment of solid malignancies including renal cell carcinoma, hepatocellular carcinoma, and thyroid cancer [157]. Sorafenib is an inhibitor of FLT3-ITD, but is not active against FLT3-TKD1 and -TKD2 mutations (Table 1 and Table 2). A phase I trial of 16 patients reported that oral administration of sorafenib reduced blast percentage in 3 FLT3 wild-type and 6 FLT3-ITD+ patients, but no response was seen in patients carrying FLT3-D835, with or without a concurrent FLT3-ITD mutation [158]. The combination of sorafenib with clofarabine and cytarabine in pediatric relapsed/refractory acute leukemia patients showed good anti-leukemic activity and tolerability [159], with 83.3% (10/12) of patients displaying a decreased blast percentage, and 6 patients (3 FLT3-ITD+, 3 FLT3 wild-type) achieving CR. In a phase I/II study, combination therapy of sorafenib with cytarabine and idarubicin in treatment-naive AML patients under 65 years, 83% of FLT3 wild-type patients and 95% of FLT3 mutant patients showed a CR or PR [160]. Responders included patients with D835 and ITD-D835 mutations, demonstrating that combination therapy may sensitize D835 mutants to sorafenib. In a randomized, double-blind phase II clinical trial, 267 treatment-naive AML patients under the age of 60 were randomized to receive induction and consolidation chemotherapy combined with either placebo or sorafenib [161]. Despite no significant difference in 3-year OS rates, patients receiving sorafenib displayed significantly increased EFS and RFS. Further analysis of OS after long-term follow up is now warranted.

Sorafenib has also been assessed in combination with the hypomethylating agent azacytidine in a phase II trial of relapsed/refractory FLT3-mutant AML [162]. Of 37 patients enrolled, 36% demonstrated a clinical response, showing promise for FLT3 inhibitor combinations beyond the highly-toxic chemotherapy regimens. Additionally, unlike chemotherapy regimens, sorafenib and azacytidine combination therapy was not associated with a high level of induced FLT3 ligand expression. In a small trial of sorafenib plus azacytidine in older FLT3-ITD+ AML patients who were unfit for chemotherapy, 78% achieved a clinical response [163]. Sorafenib and azacytidine combination therapy is currently in clinical trial for evaluation in older AML patients who are treatment naive, with interim results demonstrating significantly longer durations of remission in patients who received sorafenib compared to those receiving azacytidine only (14.5 months compared to 3.8 months, respectively) [164].

*Sunitinib* (*SU11248*): Sunitinib is an oral, multi-targeted TKI with FDA approval for treatment of metastatic renal cell carcinoma, gastrointestinal stromal tumors, and pancreatic neuroendocrine tumors [157]. Sunitinib is effective against FLT3-ITD and a subset of FLT3-TKD mutations (Table 1), with sunitinib treatment able to overcome PKC412 resistance in vitro [31,165]. In a phase I trial of 15 relapsed/refractory AML patients treated with sunitinib, patients harboring FLT3 activating mutations achieved only PR [166]. In a study of relapsed/refractory pediatric FLT3-ITD+ AML, patients were administered sorafenib combined with chemotherapy, followed by sunitinib treatment upon loss of responsivity to sorafenib [31]. Sorafenib resistance was associated with the emergence of secondary FLT3-TKD mutations, with sunitinib demonstrating efficacy against D835H and F691L point mutations, but not D835Y (Table 2). This study demonstrates how knowledge of the resistance profile of FLT3 inhibitors combined with the characterization and monitoring of FLT3 mutations throughout patient therapy, may lead to clinical benefit through sequential TKI administration.

*Lestaurtinib* (*CEP-701*): Lestaurtinib is an oral TKI that inhibits FLT3-ITD phosphorylation with an IC_50_ of 3 nM [167]. In a phase I/II clinical trial with 17 refractory/relapsed AML patients expressing FLT3-activating mutations, lestaurtinib monotherapy demonstrated minimal toxicity and led to a significant reduction in peripheral or bone marrow blasts in 5 patients. However, these responses lasted less than 3 months, possibly due to the advanced and pretreated nature of disease. Encouragingly, ex vivo drug assays demonstrated potent inhibition of FLT3 phosphorylation by lestaurtinib in samples from all patients that displayed a clinical response. Samples from 3 further patients showed reduced FLT3 activity ex vivo; however, they did not show a clinical response to lestaurtinib, suggesting reliance on alternate oncogenic signaling pathways in addition to FLT3 [168]. In a phase II trial of lestaurtinib administered as a single-agent in older patients, reduced peripheral or bone marrow blasts were seen in 60% of patients harboring FLT3-mutations and 23% of FLT3 wild-type patients. Again, clinical response correlated with drug response ex vivo. [169]. In a larger randomized phase II trial with FLT3-ITD+ AML patients in first relapse receiving chemotherapy alone or chemotherapy followed by lestaurtinib, there was no significant improvement in response rates or OS between both treatment groups. However, patients that demonstrated reduction in FLT3 phosphorylation to below 15% of baseline whilst receiving lestaurtinib displayed significantly better median survival than those that did not [170]. Together, these studies highlight the validity of targeting FLT3 signaling for treatment of AML, and suggest that monitoring FLT3 inhibition and ex vivo drug screening may help determine which FLT3 inhibitor or therapy combination to use to elicit the greatest possible patient response.

*Tandutinib* (*MLN-518*): Tandutinib is a FLT3, KIT and PDGFR TKI active against the autophosphorylation of FLT3-ITD with IC_50_ ranging from 10–100 nM [171]. In a phase I trial with tandutinib, 40 patients with AML or high-risk MDS were treated with doses ranging from 50 mg to 700 mg twice daily. Two out of five FLT3-ITD+ patients evaluable for assessment showed anti-leukemic activity of tandutinib, with a decrease in both bone marrow and peripheral blasts when treated with doses ranging from 525–700 mg. However, within two months, both patients experienced disease progression [172]. The development of resistance to tandutinib follows the same pattern of most TKIs, arising from the acquisition of an additional point mutation in the kinase domain at residue D835 [144].

#### 6.1.2. Second-Generation TKI

*Quizartinib* (*AC220*): Quizartinib is a more selective and potent FLT3 inhibitor compared to the first-generation agents (Figure 3), and therefore, displays less off-target effects; however, it is not active against a range of FLT3-TKD mutants (Table 1 and Table 2). In a phase I study of quizartinib administered daily to 76 relapsed/refractory AML patients irrespective of FLT3 status, 53% of FLT3-ITD+ patients and 14% of FLT3 wild-type patients displayed a clinical response. [173]. A phase II study assessed the efficacy and safety of two lower doses of quizartinib monotherapy in 76 FLT3-ITD+ patients with relapsed/refractory AML [174]. Patients were randomized to receive either 30 or 60 mg quizartinib daily, with 50% of patients in each group achieving composite CR (defined as complete remission + complete remission with incomplete platelet recovery + complete remission with incomplete hematological recovery) [174].

A larger-scale phase II trial of 333 relapsed/refractory AML patients has recently confirmed these response rates. Two cohorts of patients were recruited: those 60 years or older, and those 18 years or older, with 56 and 46% of FLT3-ITD+ patients achieving a composite CR in each group, respectively; and 36 and 30% of FLT3-ITD negativepatients [175]. This led to a phase III randomized controlled trial of refractory/relapsed AML, with 367 FLT3-ITD+ patients randomized to receive quizartinib or standard of care chemotherapy [176]. The use of quizartinib in this trial significantly out-performed chemotherapy, with median OS rates of 27 and 20.4 weeks, respectively. These favorable results have prompted the initiation of a phase III clinical trial evaluating quizartinib for newly-diagnosed AML in combination with standard of care (www.clinicaltrials.gov, NCT02668653). The safety of quizartinib plus chemotherapy has been demonstrated in a pilot study of 55 older patients with newly-diagnosed AML (median age of 69 years). Among the 42 evaluable patients, 33 achieved CR; including all 4 FLT3-ITD carriers [177].

*Gilteritinib* (*ASP2215*): Gilteritinib is a dual inhibitor of FLT3 and AXL. Gilteritinib is active against both FLT3-ITD and -D835 mutations, and concurrently inhibits the AXL kinase which is associated with FLT3 inhibitor resistance [178]. In a phase I–II study of 252 relapsed/refractory AML patients, gilteritinib was well tolerated, with 37% of FLT3-ITD+ patients achieving composite CR, along with 9% of FLT3 wild-type patients [179]. Of the patients that had received previous treatment with sorafenib, 49% achieved a clinical response, and 54% of patients with dual FLT3-ITD-D835 mutations achieved a composite CR, demonstrating that gilteritinib may overcome some of the acquired resistance mechanisms observed in response to preceding FLT3 TKI treatment. Following on from these promising results, a phase III trial of gilteritinib in FLT3 mutant relapsed/refractory AML is in progress [180].

*Crenolanib*: Crenolanib is an oral FLT3 TKI active against FLT3-ITD and FLT3-TKD mutations; however, its pharmacokinetics dictate drug administration multiple times a day. In a phase II trial of 34 FLT3 mutant patients, 47% achieved a clinical response [181]. In another small trial of 65 patients with refractory/relapsed AML [182], 50% of patients that had not received prior FLT3 TKI therapy achieved a clinical response. Of those that had prior exposure to FLT3 inhibitors, 31% demonstrated a clinical response. Interestingly, relapse following crenolanib treatment was not associated with the acquisition of further FLT3 mutations. Crenolanib combined with chemotherapy is currently in trial for treatment of newly diagnosed AML (www.clinicaltrials.gov; NCT02283177, NCT03258931).

## 7. Mechanisms of Relapse

### 7.1. FLT3-Mediated Mechanisms

Chemotherapy plays a significant role in disease progression in AML, instigating new mutations in founding clones or one of their subclones, which then can undergo selection and clonal expansion [7]. Chemotherapies can also select for resistant clones or LSCs (discussed above) which are present at diagnosis at very low frequencies [183]. Patients with FLT3-ITD mutation often form resistance to TKIs following the acquisition of point mutations in the activation loop of the TKD1 (i.e., N676) or TKD2 (most frequently D835), or the “gatekeeping” domain (i.e., F691) in FLT3 [183] (Table 1). These additional mutations in the FLT3 receptor (Figure 1) alter the structure and activation status of the activation loop, locking the receptor in a constitutively open conformation [184]. These mechanisms were first identified in a FLT3-ITD AML patient treated with midostaurin who developed resistance following the acquisition of a single amino acid substitution at position 676 (N676K) (Figure 1, Table 1). The substitution destabilized receptor conformation, inhibiting drug binding, and was recognized as the sole cause of resistance to midostaurin [24]. Whether this additional lesion was acquired or already present in subclones selected under treatment remains to be clearly determined, but provides a biochemical mechanism of resistance to TKIs that is now under intensive investigation. Knowledge of the sensitivities for each TKI against each FLT3 mutation is beginning to provide us with a road map for selection of the next line of therapies (Table 1 and Table 2). For example, if a FLT3-ITD AML patient develops resistance to sorafenib due to the selection of a subclone harboring a FLT3-ITD-D835 mutation, or induction of this mutation during 7 + 3, the patient is still likely to be sensitive to crenolanib or lestaurtinib (Table 2). So, TKI substitution in real-time could be effective treatment strategy. Further profiling of specific mutations and their sensitivities to TKIs is needed, which will provide us with comprehensive understanding of which mutations are sensitive to which TKIs and which inhibitors are best for each mutation or patient (Table 2).

FLT3 inhibition using small molecules is an effective treatment strategy; however, future drug development will see new TKIs targeting alternative-binding sites within FLT3 to remain active even in the presence of addition FLT3 mutations. It is interesting that in most cases of resistance, the resistance mechanism preserves FLT3 signaling; therefore, the rational design of combinations of therapies to provide therapeutic inhibition of FLT3 signaling, but to also overcome the development of resistance, is an important research endeavor, and remains the focus of research efforts worldwide.

### 7.2. Non FLT3-Mediated Mechanisms

The use of TKIs in FLT3-ITD mutant AML patients leads to the clearance of circulating blasts; however, this treatment strategy has little efficacy for blasts protected within the bone marrow niche. It is now clear that the bone marrow stroma enhances survival mechanisms by the production of signals that activate cooperative oncogenic signaling. Patients resistant to quizartinib maintain RAS/MAPK signaling downstream of FGFR1 through the increased expression of FGF2 in the bone marrow stromal cells [185]. The combination of a hypomethylating agent such as azacytidine or decitabine, with quizartinib or sorafenib, ameliorates the protection afforded by the bone marrow, inducing in vivo and ex vivo apoptosis, growth inhibition, and terminal differentiation in FLT3-ITD AML blasts [186]. Fc-optimised FLT3-antibodies are currently in clinical trial (FLYSYN-101) following promising results in a small scale patient study [187], and may present an alternative means to achieve clearance of residual blasts following front-line therapy.

Constitutive activation of FLT3 signaling through means other than mutations to the FLT3 receptor itself may also contribute to TKI resistance. Acquisition of mutations in FLT3 downstream signaling mediators, such as NRAS [145,188], may enable conservation of FLT3 signaling in the presence of FLT3 inhibitors, leading to relapse. The contribution of elevated expression of FLT3L is also an important mechanism of resistance to TKIs. FLT3L can originate in the bone marrow stroma and T lymphocytes in vivo following chemotherapy, and impairs the efficacy of TKIs [189]. ERK signaling mediates the protective effect afforded by FLT3L, with MEK inhibition now an important drug target [189]. Combinations of drugs that target mutant FLT3 and bone marrow stroma signaling are important strategies that require further investigation. It is important that these studies include analysis of cooperative and alternative signaling pathways both pre- and post-treatment to help in the prediction of which pathways may contribute to the resistance that may subsequently form. Work in this regard will help us to stratify patient treatment based on signaling pathway signatures, rather than selecting therapies purely based on genetics.

## 8. Conclusions

Landmark genomics studies have laid the foundations for the development of treatment strategies tailored to individual patients. These sophisticated studies reveal several driver mutations that are now well linked to disease development, progression, and relapse in AML. This is indeed the case for the most commonly mutated gene in AML-FLT3. Advancements in genomics technologies, coupled with access to the archival AML tissue of thousands of well-defined patients, has facilitated the unequivocal association between activating mutations in FLT3 and a poor prognosis, and high rate of relapse [9].

Since the uncovering of activating mutations to the *FLT3* gene [11] and the subsequent realization of the high frequency of these events [190,191], rapid development of broad and, more recently, selective inhibitors of FLT3, have flooded the AML clinical trials space. Both classes of inhibitors induce an initial favorable clinical response; however, the development of inhibitor resistance, followed by rapid disease relapse, occurs in almost every case. Relapse commonly develops following the acquisition of a secondary, drug resistant lesion to the *FLT3* gene, with resistance mechanisms now identified for almost all of the FLT3 TKIs in clinical use (Table 2).

The important correlation between the level of on-target inhibition (in this case, phosphorylation of FLT3) of some TKIs, and the positive clinical response seen, provides us with an opportunity to monitor treatment for patients’ in near real-time [40]. Furthermore, sophisticated and quantitative, unbiased phosphoproteomic profiling [39] now affords us with the tools necessary to uncover and monitor the key signaling pathway divergences associated with each specific FLT3 mutation, such that the design of future therapeutic approaches may hinge on the identification of activated signaling pathways rather than on somatic mutations alone. These tools are beginning to provide us with the means to position therapies for precisely the right patient, and to predict the level of treatment efficacy and durability. However, more work needs to link recurrent driver mutations and cooperating passenger lesions with their respective oncogenic signaling pathways, particularly for mutations emerging at relapse (Figure 2 and Figure 3, Table 1 and Table 2).

FLT3 inhibitor monotherapy is insufficient to achieve sustained clinical responses. This is likely due to more than one driver gene mutation being required for the development of AML. Therefore, it follows that successful AML therapy will require multifactorial gene targeting. In 2017, the FDA approved the use of four new drugs for the treatment of AML, providing hope that clinical teams will have the armory to prescribe the right combination of drugs for the right patient [192]. It is further hoped that research teams will collaborate with clinical teams to monitor on-target efficacy and determine treatment response in real-time to allow for bidirectional communication between clinicians and scientists so that dynamic drug exchange can occur before disease burden becomes too high. Among the newly FDA approved drugs is the multikinase inhibitor midostaurin, approved for administration in combination with chemotherapy in front-line treatment of FLT3 mutant AML. This development occurred on the back of the results of the RATIFY trial, which demonstrated a significant improvement in median survival for patients on midostaurin in combination with chemotherapy (74.7 months compared to 25.6 months for chemotherapy alone) [35]. This supports the notion that combination therapies may be required to achieve sustained therapeutic benefit. The toxicity of the 7 + 3 AML chemotherapeutic regimen precludes this as a treatment option for some patients, particularly the elderly. Further combinations of FLT3 inhibitors with hypomethylating agents which show a lower toxicity profile are showing promise in clinical trials [164], and need to be further investigated.

A good proportion of FLT3 wild-type patients display response to FLT3 inhibitors, suggesting that genomic profiling alone may not provide optimal patient stratification; however, little data is currently available on the role that these combinations play in controlling oncogenic signaling in FLT3 wild-type AML. Ex vivo profiling of drug response may aid in the identification of the most effective drug combination for each individual patient, and may also help to identify effective secondary therapies in the resistance setting [31,167,168,169]. However, detailed investigations into the key signaling pathway divergences linked to FLT3 mutations will hopefully provide us with a means to position therapies precisely for the right FLT3 patient subtypes (Figure 2), with future work in this space likely to uncover novel therapeutic treatment targets that will improve combinatorial targeting of mutant FLT3 in AML.

## Figures and Tables

**Table 1:** Mutations associated with resistance to FLT3 inhibitors.

FLT3 Inhibitor	Class, Type	Mutation/Amino Acid Change	Tyrosine Kinase Domain
PKC412 (Midostaurin)	First generation; Type 1	N676K [24] 672E [23] N676D [25,142] F691L, G697R [25] N676I, N676S, F691I [142]	TKD1
Sunitinib	First generation; Type 1	A627P, F691L [33] D835Y [31] Y842C [33]	TKD1 TKD2
Lestaurtinib	First generation; Type 1	A627P [33]	TKD1
Sorafenib	First generation; Type 2	F691L [31,33,142] A627P [33] D835H [31,143] D835V/F/I/del/Y/A [143] F691I, Y842N [142] Y842C [33]	TKD1 TKD2
Tandutinib	First generation; Type 2	D835Y [144]	TKD2
Quizartinib	Second generation; Type 2	F691L [31,33,73] A627P [33] D835V/F/I/del/Y [31,73,143] D835H [31] Y842C/H [73]	TKD1 TKD2
Gilteritinib	Second generation, Type 1	F961L [145,146]	TKD1
Crenolanib	Second generation, Type 1	F691L [147]	TKD1

**Table 2:** Mutation-specific response to FLT3 inhibitors: Fold change in IC50 compared to FLT3-ITD.

FLT3-Mut	Midostaurin	Sorafenib	Sunitinib	Lestaurtinib	Tandutinib	Quizartinib	Crenolanib	Gilteritinib
FLT3-ITD	1.00	1.00	1.00	1.00	1.00	1.00	1.00	1.00
D835Y	−3.00	190.91	7.22	1.14	>18.18	73.33	−1.72	−1.13
D835V	−3.73	-	13.75	-	>18.18	19.11	−3.14	-
ITD-D835Y	7.33	3000.00	31.67	−1.11	-	1000.00	1.53	1.17
ITD-D835V	1.08	780.94	3.25	-	-	468.42	1.44	-

Yellow indicates mutations showing no difference in sensitivity to TKIs compared to ITD mutations. Green indicate that mutations are more sensitive to TKI than ITD mutations. Red indicate mutations which are more resistant to TKIs than ITD mutations. All studies utilized Ba/F3 cell lines; however, methods of IC50 determination differed by study. Table created using data from references [31,143,146,149,152,153,154,155,156].

## Abbreviations

aa	Amino acid
alloHSCT	Allogeneic hematopoietic stem cell transplant
AML	Acute myeloid leukemia
CK-AML	Complex karyotype AML
CML	Chronic myeloid leukemia
CN	Cytogenetically normal
CR	Complete remission
Del	Deletion
DFS	Disease-free survival
EFS	Event-free survival
FLT3	FMS-like tyrosine kinase 3
FLT3L	FLT3 ligand
FLT3-mut	FLT3 mutation
HSC	Hematopoietic stem/progenitor cell
IL-3	Interleukin-3
Indels	Small insertions or deletions
ITD	Internal tandem duplication
LI-C	Leukemia-inducing cells
LSCs	Leukemic stem cells
MDS	Myelodysplastic syndrome
MLL-PTD	MLL-partial tandem duplication
MRD	Minimal residual disease
OS	Overall survival
PDGF	Platelet-derived growth factor
PR	Partial remission
RFS	Relapse-free survival
RTK	Receptor tyrosine kinase
TK	Tyrosine kinase
TKD	Tyrosine kinase domain
TKIs	Tyrosine kinase inhibitors
